# A new method of lower extremity immobilization in radiotherapy

**DOI:** 10.1186/1748-717X-7-27

**Published:** 2012-02-29

**Authors:** Xuhai Zheng, Tangzhi Dai, Xiaochuan Shu, Yuanxue Pu, Gang Feng, Xuesong Li, Dongbiao Liao, Xiaobo Du

**Affiliations:** 1 Department of Oncology, Mian Yang Central Hospital, Sichuan, People's Republic of China; 2 Department of Oncology, MianYang Central Hospital, Sichuan 621000, People's Republic of China

**Keywords:** Lower extremity, Immobilization, Radiotherapy

## Abstract

We developed a new method for immobilization of the fix lower extremities by using a thermoplastic mask, a carbon fiber base plate, a customized headrest, and an adjustable angle holder. The lower extremities of 11 patients with lower extremity tumors were immobilized by this method. CT simulation was performed for each patient. For all 11 patients, the device fit was suitable and comfortable and had good reproducibility, which was proven in daily radiotherapy.

## Background

Many tumors occur in the lower extremities, such as soft tissue sarcomas, bone sarcomas, and benign diseases [1-3]. Most patients with lower extremity tumors opt for radiotherapy because, compared to radical surgery, because multimodal treatments combining conservative surgery and radiation therapy achieves maximize long-term extremity function with excellent local control rates and minimal morbidity [4-7]. With the development of conformal radiotherapy, particularly the application of intensity-modulated radiotherapy, practitioners require new lower extremity immobilization methods that provide good reproducibility. No standard method has been established for lower extremity immobilization; most methods involve the use of alpha cradles, ankle casts, customized immobilization devices, or negative pressure vacuum air cushions [8,9]. We have developed a new method for immobilizing lower extremities with improved reproducibility and patient comfort.

## Methods

The Hospital Medical Research Ethics Committee approved our research in accordance with the laws and regulations in China and all the patients gave oral and written informed consent before the start of the study.

Our device includes a thermoplastic mask (Shanghai gray prescribed medical technology Co., LTD, China), a carbon fiber base plate (MedTec,USA), a customized headrest (MedTec,USA), and an adjustable angle holder (MedTec,USA). The thermoplastic mask and adjustable angle holder were remolded before use.

**Figure 1:**
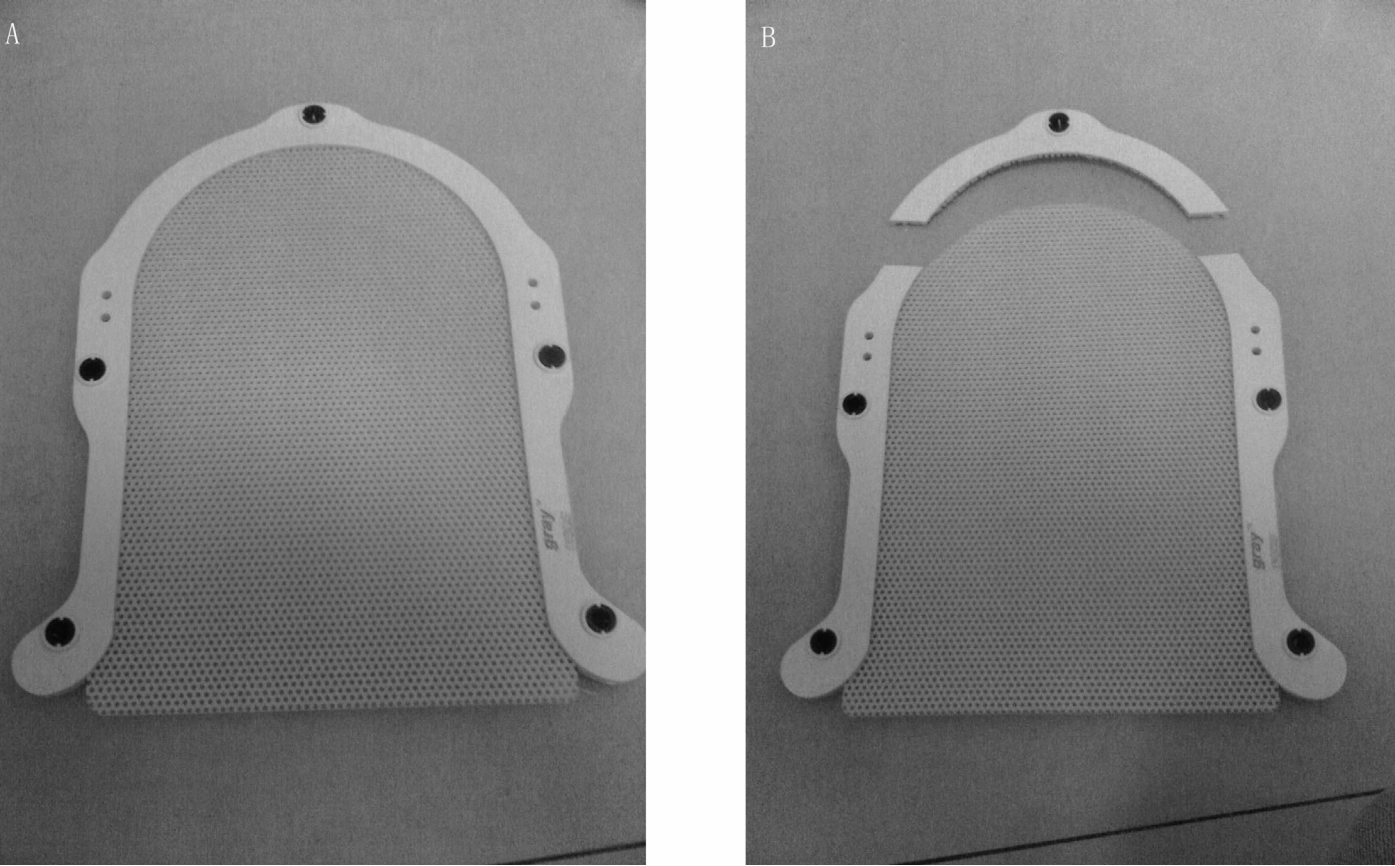
**Ready thermoplastic mask**. (A) The original thermoplastic mask for the head. (B) The mask with the rigid plastic on the top cut by us.

**Figure 2:**
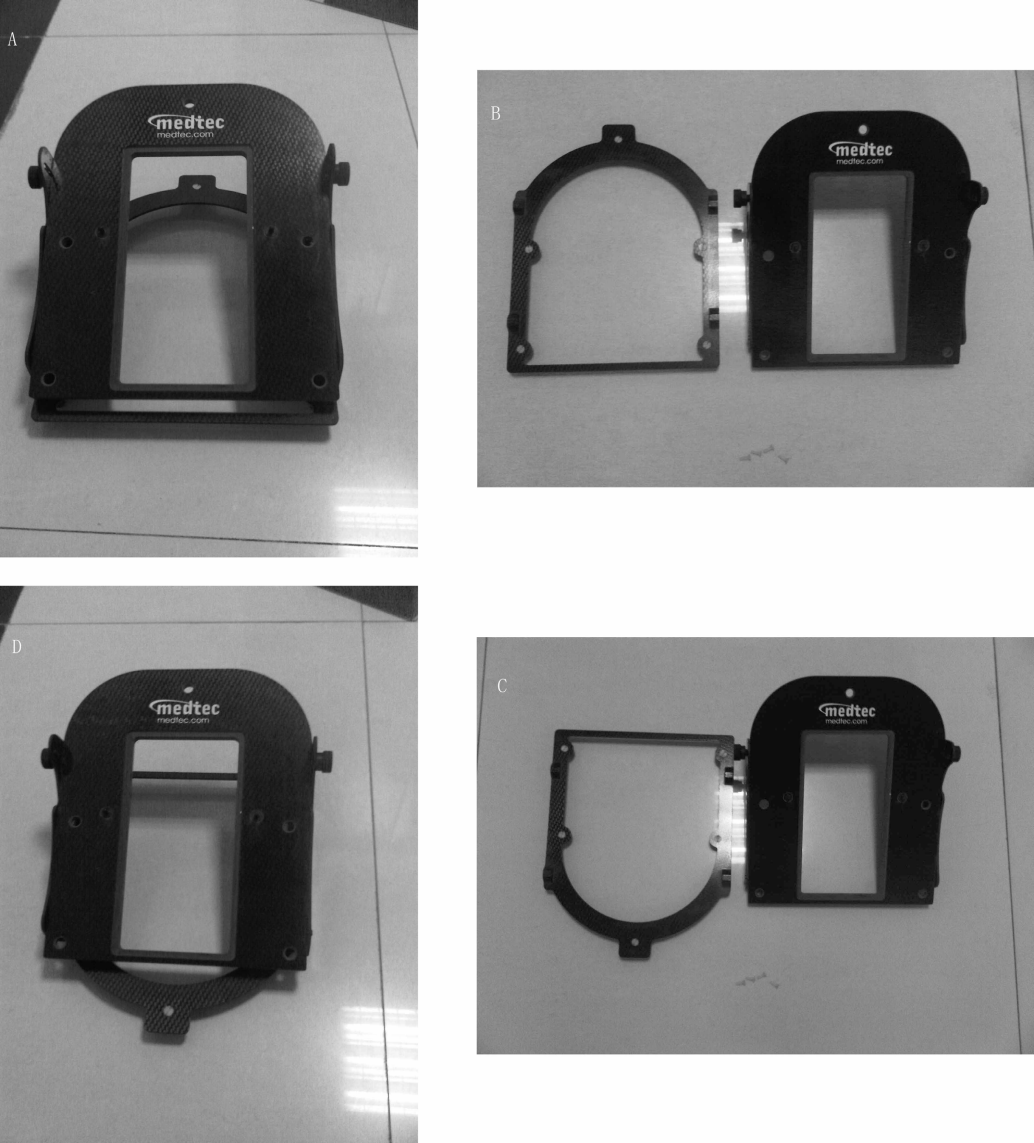
**Ready adjustable angle holder reconstructed by us**. (A) The adjustable angle holder. (B) The carbon fiber base plate at the bottom of the holder was detached by removing the 4 screws on the bottom. (C) The carbon fiber base plate was reversed. (D) The adjustable angle holder was reassembled.

**Figure 3:**
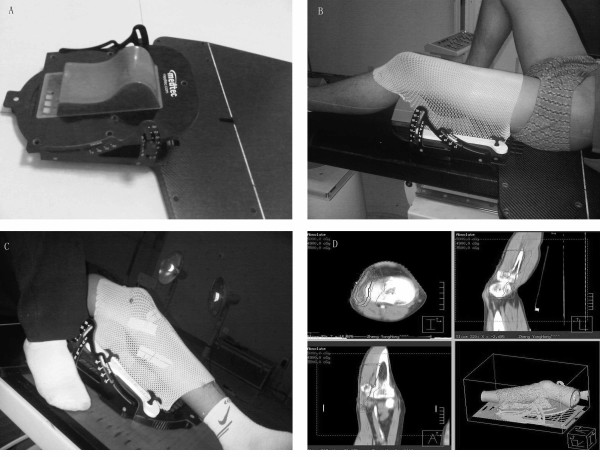
**Immobilized lower extremity**. (A) The device displayed here is a combination of the following 3 parts: the carbon base plate at the bottom, the reconstructed adjustable angle holder in the middle, and a customized headrest at the top. (B) An example of thigh fixation. (C) An example of crus fixation. (D) An image of a part of our plan system.

We cut the U-shaped thermoplastic mask (Figure 1A and 1B), removed the rigid plastic on the top, and used the remainder of the mask. We reconstructed the adjustable angle holder by detaching the carbon fiber base plate at the bottom of the holder after removing the 4 screws at the bottom, reversing the position of the plate; and fixing it again (Figure 2A, B, C and 2D). Two screws present on each side of the mask could be manipulated to change the angle for adaptation to the varying leg-bend angles. We fixed the reassembled holder to the carbon fiber base plate and placed a suitable customized headrest on the holder. Finally, we immobilized one lower extremity with the remolded thermoplastic It is important to note that only 1 leg was immobilized while the other leg was bent (Figure 3A, B and 3C).

The lower extremities of 11 patients with lower extremity tumors were immobilized by this system. CT (General Electric Company, USA) simulation was performed for each patient. For each patient, we created a plan with the Pinnacle plan system (Philips, Holland) and performed X-ray in the anterior-posterior (AP) and lateral views every weekly (Figure 3D).

## Results and discussion

The device fit for all 11 patients was suitable and comfortable, which was proven in daily radiotherapy. The mean displacement of the isocenter was 3.3 mm (from 0.4 to 6 mm) in the superior-inferior direction 2.1 mm (from 0.5 to 4 mm) in the AP direction and 1.6 mm (from 0.4 to 3 mm) in the left-right direction. The leg rotation around the isocenter was also determined and found to be negligible.

Radiotherapy plays an essential role in the treatment of lower extremity tumors [4-7,10]. In radiation methods for lower extremity tumors, Intensity-modulated radiotherapy is reported to have better target coverage than other radiation methods used for lower extremity tumors and it significantly decrease the dose to the organs-at-risk [9,11]. Intensity-modulated radiotherapy requires better reproducibility, but the lower extremities of patients with lower extremity tumors are difficult to immobilize, because of the tumor or surgical effects. In radiotherapy, immobilization of the lower extremity is achieved using alpha-cradle, ankle casts, customized immobilization devices, or negative pressure vacuum air cushions [8-10]. We developed a new method for immobilizing the lower leg by using existing materials and devices. With this method, the patients were comfortable and the reproducibility was high. First, the immobilized lower extremity could not move or turn easily, which yielded higher reproducibility. Second, some patients who were surgically treated prior to radiotherapy could not easily straighten their legs, and therefore, they found the adjustable fixation more comfortable. Third, only 1 leg was immobilized while the other leg was bent, making it easier to design treatment plans.

In conclusion, we have developed a new method for immobilizing the lower extremities for radiotherapy.

## Competing interests

The authors declare that they have no competing interests.

## Authors' contributions

XB Du undertook the conception and revision of the manuscript. XH Zheng and TZ Dai designed this study and drafted the manuscript. XC Shu and YX Pu carried out the immobilization procedure on the patients and collected the data. G Feng, XS Li and DB Liao performed data analysis. All authors read and approved the final manuscript.
